# Spatiotemporal photosynthetic physiology responses of remnant *Myricaria laxiflora* populations to regulated water level fluctuations

**DOI:** 10.1093/conphys/coaa020

**Published:** 2020-09-08

**Authors:** Shou-Peng Guan, Fang-Qing Chen, Ju-Mei Zhou, Zong-Qiang Xie, Yong-Wen Huang

**Affiliations:** 1 Hubei International Scientific and Technological Center of Ecological Conservation and Management in the Three Gorges Area, China Three Gorges University, Daxue Road 8, Yichang 443002, Hubei Province, P.R. China; 2 Engineering Research Center of Eco-environment in the Three Gorges Reservoir Region, Ministry of Education, China Three Gorges University, Daxue Road 8, Yichang 443002, Hubei Province, P.R. China

**Keywords:** Large hydropower project, *Myricaria laxiflora*, photosynthetic physiology, spatiotemporal responses, water level fluctuation

## Abstract

The construction of the Three Gorges–Gezhouba Dam cascade hydropower station has changed the water level fluctuation pattern of the habitats for remnant rare and endangered *Myricaria laxiflora* populations downstream of the dam. The present study utilized biochemical markers of photosynthetic physiology to evaluate the spatiotemporal responses of remnant populations to human-regulated water level fluctuations. The results showed that the photosynthetic physiological activities of remnant *M. laxiflora* populations underwent a period of rapid growth, followed by a gradual decline in the growth recovery phase after flooding. During the entire experimental period, photosynthetic physiological activities of remnant *M. laxiflora* populations changed with prolongation of emergence time: specifically, net photosynthetic rate and stomatal conductance initially decreased and then subsequently increased, intercellular carbon dioxide concentrations peaked at mid-phase and transpiration rate continuously increased. The maximum net photosynthetic rate, apparent photosynthetic quantum efficiency and dark respiration rate in the light–response curves of the plants continuously increased during growth. The water level gradient also significantly affected the photosynthetic physiological activities in the remnant populations, i.e. the photosynthetic physiological activities of high-altitude plants were significantly higher than the middle- and low-altitude plants. The changes in photosynthetic pigment content of plants in remnant populations during the growth recovery phase and the entire growth period were similar to those occurring in photosynthetic activities in plants. Further, canonical correspondence analysis showed that photosynthetic physiological activities in the plants were significantly correlated with changes in water levels, emergence time, elevation gradient, soil water and soil nitrogen contents. Therefore, the artificial regulation of water level fluctuations by large hydropower stations will inevitably affect the photosynthetic activities and growth of remnant *M. laxiflora* populations.

## Introduction

Water level fluctuations at riparian zones with different altitudes cause significant spatial heterogeneity in emergence time, emergence duration and soil water content (Glenz *et al*., 2006). Environmental changes caused by water level fluctuations are key factors affecting the photosynthetic physiology of riparian plants (Shen and Chen, 2015). Changes in the photosynthesis of plants occur with water level fluctuations (Kozlowski 1997; Smaoui *et al*., 2011). When water level rises and flooding occurs, photosynthetic activities of plants decrease significantly (Kristen and Brian, 2013). As the water level declines, the photosynthetic activities recover rapidly (Chen *et al*., 2005). However, when the water level retreats for a long period of time, plants are stressed by drought as groundwater remained at low levels. During such periods, the photosynthetic activities of plants are also inhibited (Wang *et al*., 2017).

Over time, riparian plants have adapted to river water level fluctuations in terms of morphology, life history, physiology and biochemistry (Blom *et al*., 1990; Li *et al*., 2015). Normal water level fluctuations generally cause fluctuations in the photosynthetic physiology of plants, but will not seriously impact the photosynthetic physiological activities during the entire growth period (Chen and Xie, 2008; Luo *et al*., 2011). Some plant species require certain water level fluctuations to promote photosynthesis (Hussner and Meyer, 2009; Bovee and Scott, 2010; Zhang *et al*., 2016). However, when changes in river water level configurations occur, the photosynthetic physiology of riparian plants is severely affected (Li *et al*., 2013; Vivian *et al*., 2014). Large water conservancy and hydropower projects have been completed for flood scarcity prevention and electricity generation; however, these efforts have also significantly changed downstream water level fluctuation patterns (Poff *et al*., 2007; Duan *et al*., 2016), which in turn affect the photosynthetic physiology of downstream riparian plants (Kitamura *et al*., 2009; Chen *et al*., 2010).

*Myricaria laxiflora* is a perennial shrub belonging to the family Tamaricaceae. *M. laxiflora* is mainly distributed in the riparian zone between Yichang at the Yangtze River and Yibin in the Jinsha River. The Three Gorges Reservoir Region is the core distribution region of this plant (Wang *et al*., 2003; Chen *et al*., 2005). The construction of the Three Gorges project has drastically raised the water level in the reservoir, flooding all habitats in the region, causing *M. laxiflora* to become an endangered species (Chen and Xie, 2009b). The Three Gorges project also changed the pattern of water level fluctuations in the riparian region from summer floods–winter emergence to summer emergence–winter floods, transforming the riverbank into an unsuitable habitat for this species (Chen *et al*., 2005; New and Xie, 2008). Conservation of the remnant populations of this species has become an inevitable option for conserving this endangered species. However, remnant *M. laxiflora* populations are located at the downstream regions of large cascade hydropower stations such as the Three Gorges–Gezhouba Dam and Xiluodu–Xiangjia Dam. The necessary regulation of the river by these large power plants for flood prevention and power generation has changed the water level fluctuation pattern in this habitat: (i) delayed the emergence period of the habitat and recovery growth phase and shortened the entire growth period and (ii) accelerated the retreat rate of water levels, reduced the water level during the dry season and decreased soil water content (Sun *et al*., 2012; Duan *et al*., 2016). Field surveys showed that population reproduction and seedling growth and regeneration of this species are dwindling and changes in water level fluctuation patterns are considered to be the key factors leading to these changes (Bao *et al*.. 2010; Chen and Wang, 2015; Chen *et al*., 2019).

We hypothesized that human-regulated water level fluctuations and resulting changes in soil and aquatic environments also significantly affect the photosynthetic physiology of remnant *M. laxiflora* populations as *M. laxiflora* plants have adapted to previous water level fluctuations to a certain degree. The photosynthetic parameters such as photosynthetic rate, stomatal conductance and the content of photosynthetic pigments of plants significantly change. To test our hypothesis, we established quadrats along the water level fluctuation gradient at the habitats of remnant populations from various elevations at different time points and measured biochemical changes related to their photosynthetic physiology. The aims of this study were to (i) measure the biochemical characteristics of photosynthetic physiology in *M. laxiflora* populations at different time points of the recovery stage and other growth stages and to analyze temporal changes in the patterns of photosynthetic parameters such as photosynthetic rate, stomatal conductance and photosynthetic pigment contents of plants with recovery growth and growth period; (2) measure the biochemical characteristics of photosynthetic physiology of *M. laxiflora* plants at different elevations and analyze spatial changes in photosynthetic parameters and photosynthetic pigment contents of plants with elevation gradient; and (iii) comprehensively analyze the effects of environmental changes in river water levels, exposure time, soil moisture and soil nutrients caused by water level fluctuations in habitat on the photosynthetic parameters and photosynthetic pigments contents of plants. This was combined with the spatiotemporal variations in artificial water level fluctuations in the habitats of remnant populations to analyze its effects on the photosynthetic physiology of plants.

## Material and methods

### Study site and quadrat setup

The study site was located at the Yanzhiba island located at Yichang at the lower reaches of the Three Gorges Dam in the main stream of Yangtze River (111°19′26″E, 30°38′57″N). The largest remnant *M. laxiflora* population is currently located at this habitat (Chen and Wang, 2015). The climate of this site is central Asian tropical monsoon, with an annual mean temperature of 16.9°C, maximum temperature of 41.4°C, minimum temperature of −9°C and annual mean rainfall of 1164.1 mm. The soil type of the site is sandy soil. The plant community at the site is *M. laxiflora* + *Salix variegata* community. In addition to the structural species, other plant species such as *Phragmites australis* and *Capillipedium assimile* are also present.

The habitat was divided into three zones including high elevational zone (≥45.1 m), middle elevational zone (45.1–42.8 m) and low elevational zone (≤42.8 m). Five 5 m × 5 m quadrats were set up at each elevational zone at 10-m intervals. Each 5 m × 5 m quadrat was used as a replicate. These quadrats were used for measurement of photosynthetic physiology in *M. laxiflora* plants and investigating changes in soil water content in the study site of the remnant *M. laxiflora* population.

### Measurement of water level changes and soil water content

The river water level report at the Yichang section in the Yangtze River Hydrology Network (www.cjh.com.cn) was used as a basis for water level changes at the study site. In addition, on-site surveys were conducted once every 3 days during the fluctuation to validate the emergence and flooding situation of the various transect lines. The emergence time of the high, middle and low elevation transect lines was November 4, 10 and 13, 2017, respectively. The submergence times were June 2, May 19 and May 2, 2018, respectively. The total emergence period was 210, 190 and 170 days, respectively.

Soil samples were simultaneously collected at the aforementioned time points during photosynthesis measurements. Random spots within the quadrats were selected, and a ring cutter was used to collect soil samples (15-cm depth). The samples were transported back to the laboratory, dried and weighed, and then soil water content was measured. Five soil samples were collected from each elevation in each emergence stage. A total of 105 soil samples were collected in the entire measurement cycle. Simultaneously, the texture and nutrient composition of soil was measured (Table 1). The composition of soil particle size was analyzed using a TopSizer laser particle size analyzer (OMEC Instruments Co., Ltd, Guangdong, China). Soil nitrogen concentrations were tested using the Kjeldahl determination, soil phosphorus concentrations tested using the method of Acid fusion-Mo sb colorimetry and the soil potassium concentrations tested using the method of NaOH melting-lame photometry.

**Table 1:** Environmental characteristics of remnant *M. laxiflora* populations distributed along elevation gradient

Elevation	Exposure date	Exposure duration (days)	Soil water content (%)	Soil texture composition and type				Soil nutrient content		
				Clay (<2 μm) (%)	Silt (2–20 μm) (%)	Sand (20–2000 μm) (%)	Soil type	Nitrogen (mg/kg)	Phosphorus (g/kg)	Potassium (g/kg)
High elevation	November 4	210	10.75 ± 1.21b	1.16 ± 0.21a	26.12 ± 4.14a	72.72 ± 4.35b	Sandy loam	55.91 ± 3.10a	0.84 ± 0.11a	20.91 ± 0.58a
Middle elevation	November 10	190	15.51 ± 0.83a	1.02 ± 0.76a	26.41 ± 1.40a	72.57 ± 1.46b	Sandy loam	46.47 ± 7.56a	0.63 ± 0.05a	20.66 ± 0.47a
Low elevation	November 13	170	6.74 ± 0.44c	0.71 ± 0.10a	13.23 ± 1.29b	86.06 ± 1.45a	Loamy sand	15.77 ± 4.15b	0.69 ± 0.09a	22.19 ± 0.53a

Note: Mean ± SE, different letters between columns indicate significant differences (*P* < 0.05). The exposure date and duration were delayed and shortened with decreases in elevation. Soil texture and soil nutrient contents changed significantly with elevation.

### Quantification of photosynthesis

Photosynthesis was measured in plants at the different elevational zones on clear days in the early emergence stage (high elevation 4 December 2017; middle elevation 10 December 2017; low elevation 13 December 2017), middle emergence stage (high elevation 12 February 2018; middle elevation 18 February 2018; low elevation 23 February 2018) and late emergence stage (high elevation 13 April 2018; middle elevation 19 April 2018; low elevation 22 April 2018) of populations to examine the effects of elevational gradient and exposure time on the photosynthetic physiology of the remnant *M. laxiflora* population. Simultaneously, photosynthesis was measured in plants at different elevational zones on clear days within the 50 days of the growth recovery phase at 10-day intervals to examine the responses of plants to rapid reduction in water levels occurring during early emergence stage. One *M. laxiflora* plant showing good growth and a plant height > 80 cm was selected from each quadrat for measurement of photosynthesis at the aforementioned time points, and five plants were selected per sample belt. As the leaves of this species are extremely small and young branchlets are green and carry out photosynthesis, we measured the photosynthesis at the top tender branches (Chen and Xie, 2009a). The Li-6400 portable photosynthesis system (Li-Cor Inc., Lincoln, NE, USA) was used to measure the net photosynthetic rate (Pn), stomatal conductance (Gs), transpiration rate (Tr) and intercellular carbon dioxide concentrations (Ci) at the branches. Three branches per plant were measured, and the mean of these measurements was used to represent the photosynthetic characteristics of that individual. During measurement, a red–blue light source was used to control the photosynthetic radiant flux density at 1200 μmol/m^2^/s, the temperature of the leaf chamber was set to 20°C and flow velocity was set to 500 μmol/s. After photosynthesis measurements, the measured branches were cut and transported to the laboratory. The branches were dried for 24 h at 60°C until constant weight then measured in terms of dry weight. Leaf water content was calculated. The dry weight of the measured branches was used as a surrogate for leaf area to calculate the photosynthetic characteristics (Chen and Xie, 2009a).

### Measurement and fitting of light response curves

Five *M. laxiflora* plants with heights > 80 cm and exhibiting good growth were selected from each sample belt for light response curve measurements on a clear day during the early, middle and late emergence stages. A red–blue light source was used the measure the net photosynthetic rate under 10 effective radiant flux densities for photosynthesis (2000, 1500, 1200, 1000, 500, 200, 100, 50, 25 and 0 μmol/m^2^/s). The temperature of the leaf chamber was set to 20°C, and flow velocity was set to 500 μmol/s. A non-rectangular hyperbolic model was used for fitting and calculation of the light response curve parameters including maximum net photosynthetic rate (Pmax), dark respiration rate (Rd), apparent quantum yield (AQY) and light compensation point (LCP).

### Quantification of photosynthetic pigments

While plant photosynthetic physiology was measured, the top green branches were randomly collected from a plant in each quadrat. There were five plant samples within each sample belt. The collected plant samples were stored in an ice box, transported back to the laboratory. Upon arrival in the laboratory, 0.20-g fresh branches were cut and placed in a mortar for homogenization using small amounts of quartz sand and 95% ethanol. Then, 10 mL of 95% ethanol was added to the homogenate, and homogenization was continued until the tissues turned white. The homogenate was topped up to 25 mL in a volumetric flask, the absorbance at wavelengths of 665 nm (A_665_), 649 nm (A_649_) and 470 nm (A_470_) were measured, and the levels of photosynthetic pigments were calculated.

Concentration of chloroplast pigments (mg/L):

C_(chlorophyll a)_ = 13.95 × A_665_–6.88 × A_649_; C_(chlorophyll b)_ = 24.96 × A_649_–7.32 × A_665_

C_(carotenoid)_ = (1000 × A_470_–2.05 × C_(chlorophyll a)_-114.8 ×  C_(chlorophyll b)_)/245

Content of chlorophyll a (Ca), chlorophyll b (Cb), carotenoid (Car) and total photosynthetic pigments (Tpp):

Content of chloroplast pigments (mg/g) = (C × Extraction volume)/Fresh weight of sample

### Data analysis

Photosynthetic physiology and photosynthetic pigment characteristic markers were used as dependent variables, and elevation and emergence time were used as independent variables for multivariate analysis of variance (MANOVA). One-way ANOVA and Duncan’s multiple comparison was then performed to determine differences in various markers among treatment levels when the factor processing effect reached significance. At the same time, Pearson correlation analysis of photosynthesis characteristics, levels of photosynthetic pigments and environmental factors was conducted to reveal the mutual relationship between photosynthesis characteristics and levels of photosynthetic pigments as well as their responses to changes in environmental factors. Data analysis was performed using SPSS 19.0 software.

## Results

### Dynamic changes in photosynthetic physiology during the recovery growth phase of plants

**Figure 1:**
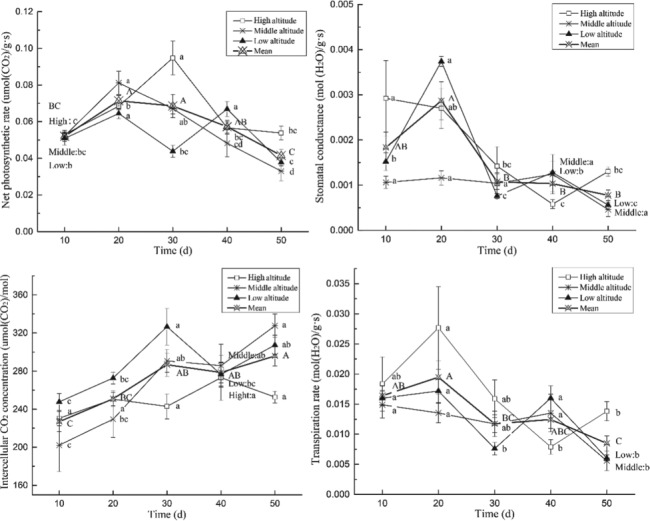
**Dynamics of the photosynthetic physiology of *M. laxiflora* plants during recovery growth.** The peak values of net photosynthetic rate, stomatal conductance and transpiration rate were observed 10–30 days after emergence of the plants, while the intercellular carbon dioxide concentrations were observed 30–50 days after emergence. Note: Different letters indicate significant differences among treatment levels. The following figures are the same

Photosynthetic physiology involves significant changes at different time points during the recovery growth stage (Pn, *F* = 10.10, *P* < 0.05; Gs, *F* = 9.89, *P* < 0.05; Ci, *F* = 7.26, *P* < 0.05; Tr, *F* = 5.59, *P* < 0.05) (Fig. 1). The Pn, Gs, Ci and Tr in plants fluctuated with variations in water level fluctuation patterns. The peak values of Pn, Gs and Tr were observed 10–30 days after emergence of the plants, and Ci reached the highest values 30–50 days after emergence of the plants, indicating that the photosynthetic physiology at 10–30 days after emergence was higher than that at 30–50 days after emergence. Elevation had some effects on the observed variations in photosynthetic physiology in plants during the experimental period, particularly the timing when peak values were observed, but not the overall variation pattern.

### Spatiotemporal changes in photosynthetic physiology during the entire growth period

The experimental period had significant effects on the Pn, Gs and Ci (*F* = 37.46, *P* < 0.05; *F* = 4.84, *P* < 0.05; *F* = 10.99, *P* < 0.05), so did the elevation (*F* = 3.29, *P* < 0.05; *F* = 3.73, *P* < 0.05; *F* = 7.30, *P* < 0.05) (Table 2). However, Tr was only significantly affected by experimental period (*F* = 4.55, *P* < 0.05). Interactions between the experimental period and elevation had no significant effects on the photosynthetic physiology of *M. laxiflora* plants (*F* = 0.37, *P* > 0.05).

**Table 2:** Univariate analysis of the effects of environmental factors on the photosynthetic physiology of *M. laxiflora* plants

Source of deviation	Dependent variable	Type III square sum	df	Mean square	*F* value	Sig.
Elevation gradient	Net photosynthetic rate	0.002	2	0.001	3.288	0.042
	Stomatal conductance	3.269E-6	2	1.634E-6	3.728	0.034
	Intercellular CO_2_ concentration	13181.468	2	6590.734	7.300	0.002
	Transpiration rate	5.664E-5	2	2.832E-5	0.370	0.694
Growth period	Net photosynthetic rate	0.036	2	0.018	37.460	0.000
	Stomatal conductance	4.240E-6	2	2.120E-6	4.835	0.014
	Intercellular CO_2_ concentration	19845.660	2	9922.830	10.990	0.000
	Transpiration rate	0.001	2	0.000	4.547	0.017
Growth period × elevation gradient	Net photosynthetic rate	0.000	4	0.000	0.207	0.933
	Stomatal conductance	1.061E-6	4	2.654E-7	0.605	0.661
	Intercellular CO_2_ concentration	960.510	4	240.128	0.266	0.898
	Transpiration rate	0.000	4	7.502E-5	0.979	0.431

Elevation gradient and growth period had significant effects on the main photosynthetic physiological activities

**Figure 2:**
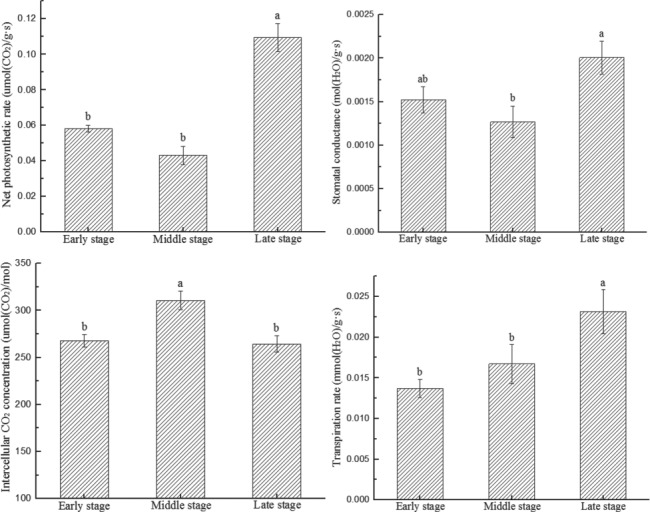
Temporal changes in the photosynthetic physiology of *M. laxiflora* plants with growth period. The net photosynthetic rate, stomatal conductance, intercellular carbon dioxide concentrations and transpiration rate changed significantly with growth period

Photosynthesis in *M. laxiflora* showed significant differences at various stages of the entire experimental period (Pn, *F* = 37.46, *P* < 0.05; Gs, *F* = 4.84, *P* < 0.05; Ci, *F* = 10.99, *P* < 0.05; Tr, *F* = 4.55, *P* < 0.05) (Fig. 2). The Pn and Gs of plants initially decreased and then subsequently increased. The Pn and Gs of plants reached the highest values of 0.1094 μmol/g s and 0.0020 mol/g s in the late experimental stage and were significantly higher than in the early and middle experimental stages (*F* = 37.46, *P* < 0.05; *F* = 4.84, *P* < 0.05), increasing by 88.3 and 153.8%, and 33.3 and 53.8%, respectively. Ci initially increased and then subsequently decreased during growth, with peak values at the middle experimental stage, which was significantly higher than in the early and middle experimental stages (*F* = 10.99, *P* < 0.05), increasing by 16.0 and 17.4%, respectively, compared to the early and late experimental stages. Tr continuously increased with experimental period, with that at the late experimental stage showing an increase of 68.6 and 38.3% (*F* = 4.55, *P* < 0.05), respectively, compared to the early and middle experimental stages.

The fitted values in the light response curve showed that the photosynthetic performance of *M. laxiflora* plants significantly changed with experimental period (Table 3). Pmax, Rd and AQY increased with experimental period as follows: early experimental stage < middle experimental stage < late experimental stage, whereas LCP decreased with emergence time as follows: early experimental stage > experimental growth stage > late experimental stage. Among these variables, the change in Pmax from the early to the middle experimental stages during growth was the highest (90.6%), whereas Pmax at the late experimental stage only increased by 14.5% relative to the middle experimental stage. Rd mainly increased from the middle experimental stage to the late experimental stage, increasing by 41.4% at the late experimental stage compared to the middle experimental stage and by 11.3% at the middle experimental stage compared to the early experimental stage. RQY also showed a greater increase at the late experimental stage (61.7%), whereas the middle experimental stage only increased by 35.0% compared to the early experimental stage. The differences in LCP at different experimental stages were not significant, with reductions of 25.3 and 23.4% being observed from the early to the middle experimental stages and from the middle to the late experimental stages, respectively.

**Table 3:** Characteristics of light response curve of *M. laxiflora* plants during different growth periods

Growth period	Pmax (μmol/g·s)	Rd (μmol/g·s)	LCP (μmol/m^2^/s)	AQY	*R* ^2^
Early growth period	0.0757	0.0115	131.42	0.0060	0.9698
Middle growth period	0.1443	0.0128	98.178	0.0081	0.9928
Late growth period	0.1652	0.0181	75.172	0.0131	0.9994

Note: Pmax, the maximum net photosynthetic rate; Rd, dark breathing rate; LCP, light compensation point; AQY, apparent quantum yield; *R*^2^, coefficient of determination. The following tables are the same.

**Figure 3:**
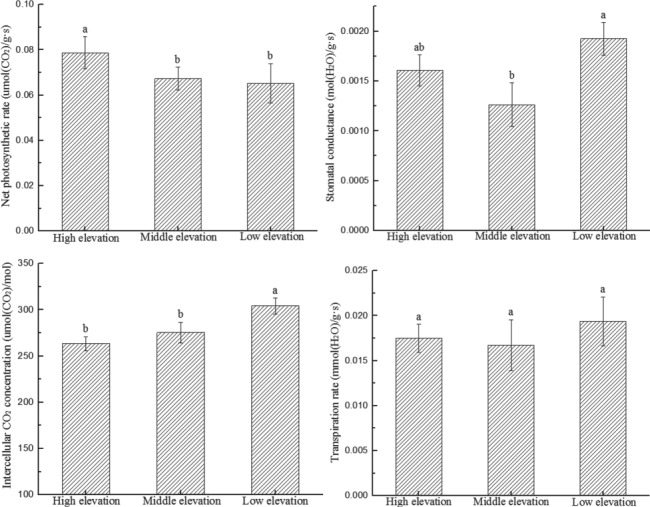
Spatial changes in the photosynthetic physiology of *M. laxiflora* plants with elevation. The net photosynthetic rate, stomatal conductance, intercellular carbon dioxide concentrations and transpiration rate of *M. laxiflora* plants changed significantly with elevation

The photosynthetic physiology of *M. laxiflora* plants showed significant spatial differences at different elevation gradients (Pn, *F* = 3.29, *P* < 0.05; Gs, *F* = 4.29, *P* < 0.05; Ci, *F* = 10.93, *P* < 0.05) (Fig. 3). Pn increased with increasing elevation. The Pn of plants at high elevation was 17.1 and 20.9% significantly higher than that at middle and low elevations (*F* = 3.29, *P* < 0.05). Gs first decreased, then increased with increasing elevation. The Gs of plants at low elevation was significantly higher than that at middle-elevation by 46.2% (*F* = 5.60, *P* < 0.05). Ci decreased with increasing elevation. The Ci of plants with low elevation were significantly higher than that at high elevation and middle elevation by 15.5 and 10.4% (*F* = 7.30, *P* < 0.05), respectively. Tr changed with elevation, although these differences were not statistically significant (*F* = 1.46, *P* > 0.05).

The fitted values in the light response curve showed that the photosynthetic performance of *M. laxiflora* changed with elevation (Table 4). Pmax and AQY decreased with elevation as follows: high elevation > middle elevation > low elevation. The Pmax of high-elevation plants was 5.8 and 28.2% higher than that of middle- and low-elevation plants, respectively. The AQY of high-elevation plants was 71.6 and 162.3% higher than that of middle- and low-elevation plants, respectively. Rd decreased with elevation as follows: middle elevation > low elevation > high elevation, and the Rd of middle-elevation plants was 37.1 and 21.4% higher than that of high- and low-elevation plants, respectively. The LCP decreased with elevation as follows: low elevation > high elevation > middle elevation and the LCP of low-elevation plants was higher than high- and middle-elevation plants by 164.0 and 39.2%, respectively.

### Spatiotemporal changes in photosynthetic pigment content in *M. laxiflora*

**Figure 4:**
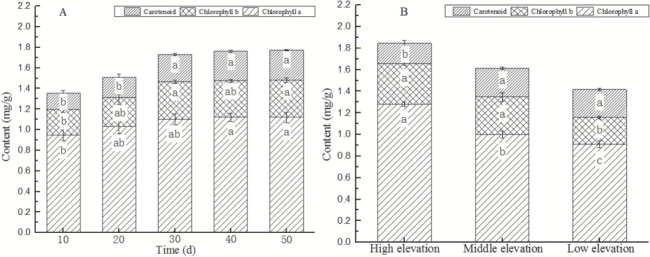
Temporal and spatial variation of photosynthetic pigments of *M. laxiflora* plants during recovering growth. Chlorophyll a and b and carotenoid contents of *M. laxiflora* plants changed significantly with recovering growth and elevation gradient

*M. laxiflora* plants showed significant changes in the levels of photosynthetic pigments at different time points during the recovery growth stage (Tpp, *F* = 6.48, *P* < 0.05; Ca, *F* = 2.83, *P* < 0.05; Cb, *F* = 2.87, *P* < 0.05; Car, *F* = 9.50, *P* < 0.05). The levels of Tpp, Ca and Cb, and Car continuously increased with recovery growth (Fig. 4A), with a greater increase in the first 30 days that plateaued off during the subsequent 20 days. The levels of Tpp, Cb and Car at Day 30 of recovery growth significantly increased by 27.2, 44.0 and 68.8% (*F* = 13.99, *P* < 0.05; *F* = 13.78, *P* < 0.05; *F* = 6.35, *P* < 0.05), respectively, compared to Day 10 of emergence.

**Table 4:** Characteristics of light response curve of *M. laxiflora* plants distributed at different elevations

Elevation	Pmax (μmol/g·s)	Rd (μmol/g·s)	LCP (μmol/m^2^/s)	AQY	*R* ^2^
High elevation	0.1338	0.0124	56.44	0.0139	0.9980
Middle elevation	0.1265	0.0170	107.05	0.0081	0.9911
Low elevation	0.1044	0.0140	148.99	0.0053	0.9842

During the recovery growth stage, the content of photosynthetic pigments in plants at different elevations also showed significant differences (Tpp, *F* = 15.79, *P* < 0.05; Ca, *F* = 43.57, *P* < 0.05; Cb, *F* = 6.40, *P* < 0.05; Car, *F* = 6.35, *P* < 0.05) (Fig. 4B). The levels of Tpp and Ca and Cb decreased with increasing elevation. The levels of Tpp and Ca and Cb in high-altitude plants was significantly higher than that of low-elevation plants by 30.3, 40.7 and 52.0% (*F* = 36.23, *P* < 0.05; *F* = 93.39, *P* < 0.05; *F* = 31.76, *P* < 0.05), respectively. However, Car decreased with increasing elevation, and the Car content of middle-elevation plants was significantly higher than that of high-elevation plants by 42.1% (*F* = 7.41, *P* < 0.05).

**Figure 5:**
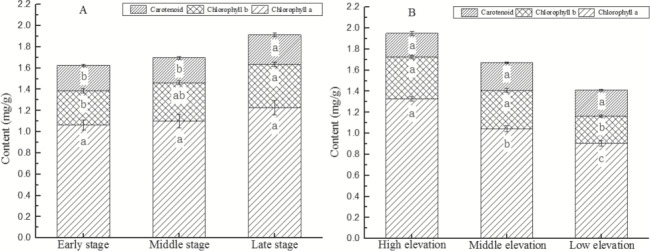
Temporal and spatial changes in photosynthetic pigments of *M. laxiflora* plants with growth period and elevation gradient. Chlorophyll a and b and carotenoid contents of *M. laxiflora* plants changed significantly with growth period and elevation gradient

The levels of photosynthetic pigments in plants showed significant differences at various experimental periods (Tpp, *F* = 4.28, *P* < 0.05; Ca, *F* = 2.02, *P* > 0.05; Cb, *F* = 4.22, *P* < 0.05; Car, *F* = 3.82, *P* < 0.05) (Fig. 5A). The levels of Tpp, Ca and Cb, and Car increased with plant growth. The levels of Tpp, Cb and Car in plants at the late experimental stage was significantly higher than at the early experimental stage by 17.2, 28.1 and 16.7% (*F* = 11.12, *P* < 0.05; *F* = 7.66, *P* < 0.05; *F* = 4.81, *P* < 0.05), respectively.

The photosynthetic pigments in plants at various elevations also show significant differences during growth (Tpp, *F* = 10.38, *P* < 0.05; Ca, *F* = 3.58, *P* < 0.05; Cb, *F* = 31.35, *P* < 0.05; Car, *F* = 2.50, *P* > 0.05) (Fig. 5B). The levels of Tpp and Ca and Cb in plants increased with increasing elevation. The levels of Tpp and Ca and Cb in high-elevation plants was significantly higher than that of low-elevation plants by 38.3, 46.2 and 53.8% (*F* = 21.68, *P* < 0.05; *F* = 5.70, *P* < 0.05; *F* = 74.76, *P* < 0.05), respectively. However, Car content decreased with increasing elevation, although these differences were not statistically significant (*F* = 2.50, *P* > 0.05).

### Analysis of the correlation between photosynthetic characteristics and pigments and environmental factors

We employed canonical correspondence analysis (CCA) to analyze the effects of environmental factors on photosynthetic physiology of the remnant population of *M. laxiflora*. The results show that the contribution rate of the principal axis I and axis II was 49.98 and 6.59%, respectively. Both axes represent 56.57% of the original information in total (Table 5). Environmental factors including river water level, elevation gradient, exposure time, soil water content, total soil nitrogen content and experimental period were significantly correlated with photosynthetic physiology (*P* < 0.01). Environmental factors had larger loads on axes 1 and 2, but the absolute value of loads on axis 2 was larger than that on axis 1.

**Table 5:** Correlation coefficients between environmental variables and CCA ordination axes

Environmental variables	CCA1	CCA2	*R* ^2^	*P*
River water level	−0.442	−0.897	0.734	0.001^***^
Elevation gradient	0.468	−0.884	0.372	0.001^***^
Exposure time	0.474	−0.880	0.377	0.001^***^
Soil water content	0.512	−0.859	0.327	0.001^***^
Total soil nitrogen	0.641	−0.767	0.431	0.001^***^
Growth period	−0.649	−0.761	0.589	0.001^***^
Cumulative contribution rate (%)	49.98	56.57		

Note: *R*^2^ represents the coefficient of determination of environmental factors on photosynthetic physiology; *P* indicates the significance of correlation analysis. ^***^P < 0.001.

**Figure 6:**
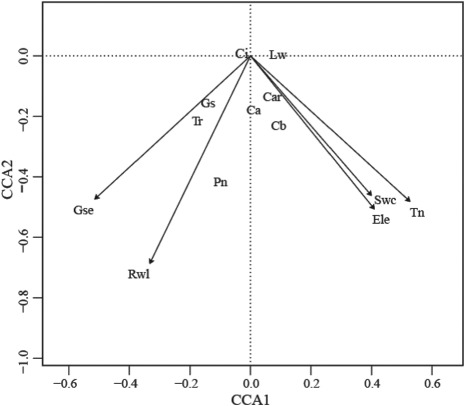
CCA ordination of plant photosynthetic physiology and environmental factors. The photosynthetic physiology of the remnant *M. laxiflora* population was influenced by changes of various environmental factors caused by water level fluctuation of the Yangtze River. Note: Pn, Note: Pn, net photosynthetic rate; Gs, stomatal conductance; Ci, intercellular CO_2_ concentration; Tr, transpiration rate; Ca, chlorophyll a content; Cb, chlorophyll b content; Car, carotenoid content; Lwc, leaf water content; Rwl, river water level; Eg, elevation gradient; Et, exposure time; Swc, soil water content; Snc, soil nitrogen content; Gp, growth period

Further CCA sequencing analysis indicated that the effects of environmental factors on photosynthetic physiology varied among photosynthetic physiological indicators (Fig. 6). Pn showed the longest projection distance (from the projection point to the central point) for river water level, while Gs and Tr showed the longest projection on growth period. Additionally, Ca had the greatest effect on river water level and elevation gradient, while Cb and Car had the greatest effect on elevation gradient, exposure time and soil water content. Finally, leaf water content had the greatest effect on total soil nitrogen content. These findings indicated that the Pn, Gs and Tr were susceptible to river water level and time of exposure to the Yangtze River, while chlorophyll content was susceptible to elevation gradient of fluctuation zone and soil water content, and leaf water content was greatly affected by total soil nitrogen content. Because all environmental factors including water level, elevation, soil water content, total soil nitrogen content and exposure time were associated with water level fluctuation, the photosynthetic physiology of the remnant *M. laxiflora* population was influenced by water level fluctuations of the Yangtze River.

## Discussion

### Temporal variations in photosynthetic physiology of *M. laxiflora* plants

Photosynthetic performance in plants often changes with the process of plant growth and development (Zegadalizarazu and Monti, 2013). Plants exhibit compensatory growth responses once a stressor is removed. Smaoui *et al*. (2011) examined the tolerance of *Cotula coronopifolia* to flood stress. They found that the chlorophyll content of these plants significantly increased after a flood for a short period of time, with chlorophyll a and b levels increasing by 167 and 295%, respectively, to rapidly increase photosynthetic rates for compensatory growth. The habitat of *M. laxiflora* experiences long periods of flooding every summer, and the leaves and young stems of these plants often completely shed during this period. The plants start regrowth when the habitat emerges after water levels had receded. Our previous study showed that the stems and leaves of this species undergo rapid growth during the early stage of recovery (Chen and Xie, 2009b). The present study used the perspective of photosynthetic physiology to assess changes in the photosynthetic performance of plants during the early stage of recovery. We found that the Pn and other photosynthetic physiology markers rapidly increase during recovery growth, which may be associated with compensatory growth after flood stress was removed. As plants consumed large amounts of energy in maintaining vitality during the flood season, they are in a ‘hunger’ state. At the recovery growth stage after flood stress was removed, only by rapidly increasing photosynthetic capacity for photosynthesis could plants compensate for nutrient deficiencies and accumulate substances and energy for later growth processes (Chen *et al*., 2010). However, plants at various elevations have differences in photosynthetic physiological compensatory growth as growth in high-elevation plants is greater than middle- and low-elevation plants.

There are usually great differences in photosynthetic physiology among plants at different growth and development stages (Zegadalizarazu and Monti, 2013; Christos and George, 2016). Riparian plants start to undergo recovery growth after their emergence. As emergence time and plant growth are prolonged, leaf tissues gradually mature, pigment content increases, enzymatic activities increase and photosynthetic capacity increases (Thomas 2010; Luo *et al*,. 2011). Substances and energy produced and stored by exuberant photosynthesis enables the plants to respond to the next flood stress (Christos and George 2016; Yang and Li 2016; Wen *et al*., 2017). The Pmax and photosynthetic pigment content of *M. laxiflora* plants continuously increase as emergence time prolongs and plants grow, showing that their photosynthetic performance continuously increases with growth. However, actual measurements show that the Pn and Gs during the middle stage of growth of *M. laxiflora* plants were lower than the early stage, indicating that their actual photosynthetic performance is lower. One explanation for these observations is compensatory growth, which increases the photosynthetic performance of plants during the early emergence stage. Another plausible explanation is that the middle emergence stage occurs in winter, and low temperatures during this time slow down plant growth and related physiological activity. Another may be related to the rapid decrease in water levels in the habitat during the middle stage. The rapid decline in water level causes soil water content in the habitat to be at the lowest level. Insufficient soil water content affects photosynthetic performance (Zegadalizarazu and Monti, 2013; Vivian *et al*., 2014). Chen *et al*. (2013) studied the photosynthetic physiology responses of a riparian plant, *Populus euphratica*, to groundwater levels. They found that when groundwater levels were lower than the normal water levels, the water potential and stomatal conductance in leaves decreased, which in turn significantly reduced photosynthesis in plants.

### Characteristics of spatial changes in the photosynthetic physiology of *M. laxiflora* plants

Spatial heterogeneity in the environment often causes changes of plants in photosynthetic physiology (Zheng and Shangguan, 2007; Gratani *et al*., 2012). Spatial changes in the photosynthetic physiology of riparian plants distributed at different elevations occur as responses to environmental heterogeneity during the exposure period, duration and soil moisture and nutrients caused by water level fluctuations (Steed *et al*., 2002; Shen and Chen, 2015). Optimal emergence duration and emergence time can significantly promote the photosynthetic physiology of plants (Wen *et al*., 2017). The emergence duration and emergence time of high-elevation habitats are earlier and longer than low-elevation habitats. Therefore, plants located at high-elevation regions have generally higher photosynthetic rates than low-elevation plants (Poff *et al*., 2007; Zhang *et al*., 2018). In addition, soil water saturation, severe water and soil nutrients deficiency caused by water level fluctuations also adversely affect the photosynthetic physiology of plants (Yang and Li, 2016). Shen and Chen (2015) found that the photosynthetic physiology of *Cynodon dactylon* in the riparian zone of the Three Gorges Reservoir Region changes with elevation, i.e. community photosynthetic characteristics and productivity were highest in 160–170-m high-elevation regions, which have significantly higher photosynthetic performance compared with middle- and low-elevation communities in the riparian zone.

The photosynthetic physiology of *M. laxiflora* show significant spatial differences as Pmax and Pn increases with higher elevation. The differences in photosynthetic physiology and photosynthetic performance of plants among various elevations originate from differences in the emergence duration, emergence time, soil water content and soil nutrients. The emergence time and duration of remnant *M. laxiflora* populations is thus earlier and longer, and the total soil nitrogen and phosphorus content increases with elevation. High-elevation plants showed faster recovery growth, longer recovery growth stage, better growth status than low-elevation plants and better photosynthetic performance. Plant photosynthetic performance is positively correlated with pigment content (Zheng and Shangguan, 2007). This is because an increase in the content of photosynthetic pigments facilitates the capture, allocation, transmission and conversion of light energy by leaves. The major photosynthetic physiology markers of *M. laxiflora* plants also showed a similar correlation with the levels of photosynthetic pigments. The levels of photosynthetic pigments and photosynthetic performance exhibited identical spatial variation patterns, i.e. high elevation > middle elevation > low elevation. The difference in photosynthetic performance among elevation gradients is because of the spatial heterogeneity of the environment caused by water level fluctuations. However, the dominant environmental factors influencing each photosynthetic physiological parameter differed from each other.

### Integrated effects of changes in water level fluctuations in the habitat on photosynthetic physiology of *M. laxiflora*

Riparian plants have developed mechanisms to adapt river water fluctuations (Glenz *et al*., 2006; Song *et al*., 2016). Although floods and droughts caused by normal water level fluctuations affect the fluctuations and changes in photosynthetic physiological activities in plants to some extent, plants can adapt to these environmental stresses through morphological and physiological changes, so that photosynthetic physiological activities are slightly altered and then subsequently restored to normalcy (Kozlowski 1997). However, when large changes in river water fluctuation patterns occur, the degree of environmental stress exceeds the adaptation range of plants. This greatly affects photosynthetic physiology in plants, which would further influence plant growth (Hussner and Meyer, 2009; Yang *et al*., 2018). Yang *et al*. (2018) found that when bitter nightshade (*Solanum dulcamara*) experiences flood stress, these form aerenchymatous adventitious roots on their stems to decrease stress damage. However, when great changes in the environment occur, resulting in complete submergence, the adventitious roots are damaged and photosynthetic physiology decreases. Changes in water level fluctuations may also affect the growth and development of plants, possibly threatening their survival, thus endangering species (Wang *et al*., 2003; Chen *et al*., 2005). Kitamura *et al*. (2009) found that after *Schoenoplectus gemmifer* has experienced several large floods, and its population size is now only one-tenth of that in the past, and their risk for extinction has significantly increased.

CCA showed that the photosynthetic activity of this species is significantly influenced by the water level of the Yangtze River, soil water content, altitude, emergence time and soil nutrients. Therefore, changes in the water level of the Yangtze River, emergence time, soil water content and soil nutrients caused by changes in water level fluctuation patterns will affect photosynthetic physiology in plants. The construction of the large Three Gorges–Gezhouba Dam cascade hydropower station has changed the water level fluctuation pattern of the habitat of remnant *M. laxiflora* populations. The emergence time of the habitat was delayed from the beginning of September to the early-mid October, while the flood period now occurs earlier, which was originally from late May to mid or even early May. The entire emergence period was shortened from 200–240 to 170–210 days. Therefore, the photosynthetic physiological activities and growth time of *M. laxiflora* plants are delayed and shortened. At the same time, during habitat emergence, the rate water level decline also increased. The timing of minimum runoff occurs earlier, from January–March to December to February of the subsequent year (Duan *et al*., 2016). The water level of the habitat during the dry period significantly decreased by around 1.29 m (Sun *et al*., 2012). The accelerated decline in water level and decline in water level during the dry period causes the soil water content in the habitat to decrease, thereby affecting the photosynthetic physiology of plants. We previously measured the photosynthetic physiology of *M. laxiflora* plants during autumn and spring in the following year under normal water level fluctuations, and the net photosynthesis intensity was 0.066 and 0.151 μmol/g·s (Chen and Xie, 2009a). These values were both higher than the Pn measured in this study at the same time range. Therefore, the construction of a large hydropower station has changed the water level fluctuation pattern of the habitat for remnant *M. laxiflora* populations. Due to the shortening of the photosynthesis period and the decrease in the photosynthetic physiology intensity in plants, the assimilation and growth of plants during the entire experimental period are further affected.

Changes in river water level fluctuations and changes in the ecological environment caused by it often affect plant photosynthetic physiology, growth and development and population regeneration (Vivian *et al*., 2014; Campbell *et al*., 2016). Bao *et al*. (2010) examined the population dynamics of remnant *M. laxiflora* populations and found that the number of seedlings is low and growth is delayed, resulting in difficulties in population regeneration and the population is at a degenerate state. Our previous study showed that soil water content has important effects on the germination of *M. laxiflora* seeds and seedling growth, while changes in river water level fluctuations affect the regeneration of the population (Yuan *et al*., 2008). The results of this study showed that the photosynthetic physiology of remnant *M. laxiflora* populations is significantly influenced by the water level of the Yangtze River, soil water content and emergence time. Therefore, artificial regulation of water level fluctuations in the Yangtze River will cause changes in the water level fluctuation configuration of the habitat of remnant *M. laxiflora* populations and severely affect photosynthetic physiology in plants. To ensure the normal operation of photosynthetic physiology in remnant *M. laxiflora* populations, it is extremely important to satisfy the aquatic environmental conditions of plants. Appropriate adjustments to the current flow regulation plan in large hydropower stations could be made so that the habitat can emerge earlier and water level decline can be slowed to satisfy the photosynthetic physiology requirements of plants.

## Conclusion

The construction of the Three Gorges–Gezhouba Dam cascade hydropower station known as the Three Gorges project has altered the pattern of water level fluctuations in the habitat of remnant *M. laxiflora* populations downstream of the dam. To uncover influences of the changes in water level fluctuations on species conservation, the physiological responses of *M. laxiflora* to regulated regimes were examined by in situ testing of the photosynthetic characteristics of populations distributed along elevation gradient during different emergence stages. Significant spatiotemporal dynamics of plants in photosynthetic physiology were observed. The photosynthetic physiological activities of remnant populations generally increased with the prolongation of emergence time throughout the experimental growth period after emergence. The fluctuation gradient also had a significant effect on photosynthetic physiological activities of remnant populations. The photosynthetic physiological activities of high-altitude plants were significantly higher than the middle- and low-elevation plants. The variations in photosynthetic pigment content of young shoots and leaves were the same as the photosynthetic physiological activities. Most photosynthetic physiological parameters in the plants were significantly correlated to photosynthetic pigment content, altitude, changes in water levels and emergence time and soil water content. The altered water level fluctuation pattern caused by the Three Gorges Dam–Gezhouba cascade water conservancy and hydropower project delays emergence time, shortens growth period, accelerates the decline rate of water level and reduces soil water content, which impacts the photosynthetic physiology of remnant *M. laxiflora* populations.

## References

[ref1] Bao DC, Lu ZJ, Jiang MX, Xu SD, Yao Q, Liu QF (2010) Structure and dynamics of remanent populations of Myricaria laxiflora in the lower reaches of the three gorges dam. J Wuhan Bot Res 28: 711–717.

[ref2] Blom CWPM, Bögemann GM, Laan P, Sman AJMVD, Steeg HMVD, Voesenek LACJ (1990) Adaptations to flooding in plants from river areas. Aquat Bot 38: 29–47.

[ref3] Bovee KD, Scott ML (2010) Implications of flood pulse restoration for populus regeneration on the upper Missouri River. River Res Appl 18: 287–298.

[ref4] Campbell D, Keddy PA, Broussard M, Mcfalls-Smith TB (2016) Small changes in flooding have large consequences: experimental data from ten wetland plants. Wetlands 36: 457–466.

[ref5] Chen FQ, Xie ZQ (2008) Physiological and biochemical characteristics of endangered plant *Myricaria laxiflora* in Three Gorges Reservoir Area. Guihaia 28: 367–372.

[ref6] Chen FQ, Xie ZQ (2009a) The physiological and biochemical responses of endangered plant *Myricaria laxiflora* to simulated summer flooding. J Trop Sub Bot 17: 249–253.

[ref7] Chen FQ, Xie ZQ (2009b) Survival and growth responses of *Myricaria laxiflora* seedlings to summer flooding. Aquat Bot 90: 333–338.

[ref8] Chen FQ, Guaan SP, Ma YR, Xie ZQ, Lv K, Huang YW, Jia GM (2019) Impact of regulated water level fluctuations on the sexual reproduction of remnant Myricaria laxiflora populations. Glob Ecol Conser 18: e00628.

[ref9] Chen FQ, Huang YZ, Fan DY, Xie ZQ (2010) Physiological and ecological effects of flooding on vegetatively propagated plants of *Cynodon dactylon*. Guihaia 30: 488–492.

[ref10] Chen FQ, Wang CH (2015) Ecological protection of the rare and endangered plant Myricaria laxiflora in the Three Gorges. Science Press, Beijing.

[ref11] Chen FQ, Xie ZQ, Xiong GM, Liu YM, Yang HY (2005) Reintroduction and population reconstruction of the endangered plant *Myricaria laxiflora* in the Three Gorges. Acta Ecol Sin 25: 1811–1817.

[ref12] Chen YN, Zhou HH, Chen YP (2013) Adaptation strategies of desert riparian forest vegetation in response to drought stress. Ecohydrology 6: 956–973.

[ref13] Christos C, George G (2016) Photosynthesis in developing leaf of juveniles and adults of three Mediterranean species with different growth forms. Photosynthesis Res 130: 1–18. 10.1007/s11120-016-0276-4 27220729

[ref14] Duan WX, Guo SL, Wang J (2016) Analysis of the influence of large reservoirs in the upper reaches of the Yangtze River on the hydrological situation of Yichang station. Resour. Environ. Yangtze Basin 25: 120–130.

[ref15] Glenz C, Schlaepfer R, Iorgulescu I, Kienast F (2006) Flooding tolerance of central European tree and shrub species. Forest Ecol Manag 235: 1–13.

[ref16] Gratani L, Catoni R, Pirone G, Frattaroli AR, Varone L (2012) Physiological and morphological leaf trait variations in two apennine plant species in response to different altitudes. Photosynthetica 50: 15–23.

[ref17] Hussner A, Meyer C (2009) The influence of water level on the growth and photosynthesis of *Hydrocotyle ranunculoides* L. fil. Flora 204: 755–761.

[ref19] Kitamura K, Jin Y, Tainaka KI, Yokojima S (2009) Potential impacts of flooding events and stream modification on an endangered endemic plant, *Schoenoplectus gemmifer* (cyperaceae). Ecol Res 24: 533–546.

[ref20] Kozlowski TT (1997) Responses of woody plants to flooding and salinity. Tree Physiol 17:490–490.

[ref20a] Kristen AP, Brian RM (2013) Effects of flooding on photosynthesis and root respiration in saltcedar (Tamarix ramosissima), an invasive riparian shrub. Environ Exp Bot 189: 19–27.

[ref21] Li J, Yu B, Zhao C, Nowak RS, Zhao Z, Sheng Y (2013) Physiological and morphological responses of *Tamarix ramosissima* and *Populus euphratica* to altered groundwater availability. Tree Physiol 33: 57–68. 23243028 10.1093/treephys/tps120

[ref22] Li ZJ, Fan DY, Chen FQ, Yuan QY, Chow WS, Xie ZQ (2015) Physiological integration enhanced the tolerance of *Cynodon dactylon* to flooding. Plant Biol 17: 59–465. 25557716 10.1111/plb.12254

[ref23] Luo FL, Nagel KA, Scharr H, Zeng B, Schurr U, Matsubara S (2011) Recovery dynamics of growth, photosynthesis and carbohydrate accumulation after de-submergence: a comparison between two wetland plants showing escape and quiescence strategies. Ann Bot 107: 49–63. 21041230 10.1093/aob/mcq212 PMC3002471

[ref24] New T, Xie Z (2008) Impacts of large dams on riparian vegetation: applying global experience to the case of China’s Three Gorges Dam. Biodivers Conserv 17: 3149–3163.

[ref25] Poff NL, Olden JD, Merritt DM, Pepin DM (2007) Homogenization of regional river dynamics by dams and global biodiversity implications. Proc Natl Acad Sci USA 104: 5732–5737. 17360379 10.1073/pnas.0609812104 PMC1851560

[ref26] Shen JE, Chen FQ (2015) Spatial and temporal dynamics of *Cynodon dactylon* in photosynthesis and productivity on riverbanks in Xiangxi River in the Three Gorges Reservoir Area In Chen S. et al. eds, Advances in Engineering Research: Vol. 23 International Conference on Advances in Energy, Environment and Chemical Engineering (2015AEECE), Atlantis Press, Changsha, China pp. 358–362. doi:10.2991/aeece-15.2015.83.

[ref27] Smaoui A, Jouini J, Rabhi M, Bouzaien G, Albouchi A, Abdelly C (2011) Physiological and anatomical adaptations induced by flooding in *Cotula coronopifolia*. Acta Biol Hung 62: 182–193. 21555270 10.1556/ABiol.62.2011.2.8

[ref28] Song Y, Ke X, Liu W, Davy AJ, Liu G (2016) Life-history plasticity of riparian annual plants adapted to extreme variations in water level: mesocosm experiments. River Res Appl 31: 1311–1318.

[ref29] Steed JE, Dewald LE, Kolb TE (2002) Physiological and growth responses of riparian sedge transplants to groundwater depth. Int J Plant Sci 163: 925–936.

[ref30] Sun ZD, Huang Q, Opp C, Hennig T, Marold U (2012) Impacts and implications of major changes caused by the Three Gorges Dam in the middle reaches of the Yangtze river, China. Water Resour Manag 26: 3367–3378.

[ref31] Thomas SC (2010) Photosynthetic capacity peaks at intermediate size in temperate deciduous trees. Tree Physiol 30: 555–573. 20335160 10.1093/treephys/tpq005

[ref33] Vivian LM, Godfree RC, Colloff MJ, Mayence CE, Marshall DJ (2014) Wetland plant growth under contrasting water regimes associated with river regulation and drought: implications for environmental water management. Plant Ecol 215: 997–1011.

[ref34] Wang Y, Wu JQ, Tao Y, Li ZZ, Huang HW (2003) Study on the natural distribution and ex situ conservation of the endemic plant *Myricaria laxiflora* in the Three Gorges Reservoir Area. J Wuhan Bot Res 21: 415–422.

[ref34a] Wang HZ, Han L, Xu YL, Niu JL, Yu J (2017) Effects of soil water gradient on photosynthetic characteristics and stress resistance of Populus pruinosa in the Tarim Basin, China. Acta Ecol Sin 37: 432–442.

[ref35] Wen BL, Li XY, Yang F, Lu XR, Li XJ, Yang FY (2017) Growth and physiology responses of *Phragmites australis* to combined drought-flooding condition in inland saline-alkaline marsh, Northeast China. Ecol Eng 108: 234–239.

[ref35a] Yang X, Jansen MJ, Zhang Q, Sergeeva L, Ligterink W, Mariani C (2018) A disturbed auxin signaling affects adventitious root outgrowth in solanum dulcamara under complete submergence. J Plant Physiol 224–225: 11–18. 10.1016/j.jplph.2018.03.006 29574325

[ref36] Yang Y, Li C (2016) Photosynthesis and growth adaptation of *Pterocarya stenoptera*, and *Pinus elliottii*, seedlings to submergence and drought. Photosynthetica 54: 120–129.

[ref36a] Yuan WQ, Zhan HY, Chen FQ, Xia HW, Luo YC, Liu CC (2008) Ecological characteristics of seed germination of endangered plant Myricaria laxiflora. Ecol Environ 17: 2341–2345.

[ref37] Zegadalizarazu W, Monti A (2013) Photosynthetic response of sweet sorghum to drought and re-watering at different growth stages. Physiol Plantarum 149: 56–66. 10.1111/ppl.12016 23198740

[ref38] Zhang AY, Fan DY, Li Z, Xiong GM, Xie ZQ (2016) Enhanced photosynthetic capacity by perennials in the riparian zone of the Three Gorges Reservoir Area, China. Ecol Eng 90: 6–11.

[ref39] Zhang W, Chen JQ, Liu J, Chen FQ, Zhang QF, Ouyang ZT, Shao CL, Fan Y, Xu WL (2018) Spatiotemporal variations of CO^2^ fluxes in a Cynodon-dominated riparian zone of the Three Gorges Reservoir (TGR). China J Plant Ecol 11: 877–886.

[ref40] Zheng SX, Shangguan ZP (2007) Spatial patterns of photosynthetic characteristics and leaf physical traits of plants in the Loess plateau of China. Plant Ecol 191: 279–293.

